# Assessment of Male Reproductive Aging Implications of Sertoli Cell’s Senescence

**DOI:** 10.1093/geroni/igaf122.2877

**Published:** 2025-12-31

**Authors:** Elikanah Olusayo Adegoke, Alexandra Montesinos, Reema Banarjee, Amit Dey, Nathan Basisty

**Affiliations:** National Institute on Aging, Baltimore, Maryland, United States; National Institute on Aging, Baltimore, Maryland, United States; National Institute on Aging, Bethesda, Maryland, United States; National Institute on Aging, Bethesda, Maryland, United States; National Institute on Aging, Bethesda, Maryland, United States

## Abstract

Advances in cancer detection and improved treatment strategies have markedly increased survival rate for cancers (pediatric), leading to a growing population of early life cancer survivors. However, long term reproductive effects of chemotherapy (doxorubicin) and radiotherapy (irradiation) on reproductive aging has received less attention. Sertoli cells support, nourish, and protect spermatogenic cells in the testis. To evaluate the senescence inducing potential of doxorubicin and irradiation on Sertoli cells and the consequent implications in male reproductive aging, Sertoli cells in culture were exposed to doxorubicin and gamma radiation, and senescence was validated ten days after exposure by confirming the upregulation of senescence markers: CDKN2A, CDKN1A, GDF15, IL-6, IL-8. DIA proteomic analysis was conducted on the Orbitrap mass spectrometer. Data analysis was performed on Spectronaut and R. The proteomic analysis identified 6,304 proteins. Among these, 1,509 proteins were downregulated and 1,443 were upregulated in doxorubicin treatment. After irradiation, 1,090 proteins were downregulated, while 1,125 proteins were upregulated. Microtubule-based process, cell division, and sexual reproduction biological processes were enriched among downregulated proteins following both treatments, suggesting loss of key functions supporting reproduction and phagocytosis associated with Sertoli cells. Furthermore, transmembrane/cell transport pathways and immune response, were enriched among upregulated proteins in both treatments, known senescence associated pathways that are consistent with dysregulation of normal Sertoli cell paracrine functions. 45% of protein changes were overlapping between both treatments. In conclusion, we demonstrate that both doxorubicin and irradiation can induce Sertoli cells to senescence and consequently result in reproductive aging marked by decreased sexual reproduction.

